# Evaluation of HLA typing content of next-generation sequencing datasets from family trios and individuals of arab ethnicity

**DOI:** 10.3389/fgene.2024.1407285

**Published:** 2024-05-27

**Authors:** Mohammed Dashti, Md Zubbair Malik, Rasheeba Nizam, Sindhu Jacob, Fahd Al-Mulla, Thangavel Alphonse Thanaraj

**Affiliations:** Department of Genetics and Bioinformatics, Dasman Diabetes Institute, Kuwait City, Kuwait

**Keywords:** HLA typing, consistency, resolutions, reproducibility, next-generation sequencing

## Abstract

**Introduction:** HLA typing is a critical tool in both clinical and research applications at the individual and population levels. Benchmarking studies have indicated HLA-HD as the preferred tool for accurate and comprehensive HLA allele calling. The advent of next-generation sequencing (NGS) has revolutionized genetic analysis by providing high-throughput sequencing data. This study aims to evaluate, using the HLA-HD tool, the HLA typing content of whole exome, whole genome, and HLA-targeted panel sequence data from the consanguineous population of Arab ethnicity, which has been underrepresented in prior benchmarking studies.

**Methods:** We utilized sequence data from family trios and individuals, sequenced on one or more of the whole exome, whole genome, and HLA-targeted panel sequencing technologies. The performance and resolution across various HLA genes were evaluated. We incorporated a comparative quality control analysis, assessing the results obtained from HLA-HD by comparing them with those from the HLA-Twin tool to authenticate the accuracy of the findings.

**Results:** Our analysis found that alleles across 29 HLA loci can be successfully and consistently typed from NGS datasets. Clinical-grade whole exome sequencing datasets achieved the highest consistency rate at three-field resolution, followed by targeted HLA panel, research-grade whole exome, and whole genome datasets.

**Discussion:** The study catalogues HLA typing consistency across NGS datasets for a large array of HLA genes and highlights assessments regarding the feasibility of utilizing available NGS datasets in HLA allele studies. These findings underscore the reliability of HLA-HD for HLA typing in underrepresented populations and demonstrate the utility of various NGS technologies in achieving accurate HLA allele calling.

## Introduction

The human leukocyte antigen (HLA) complex, a key component of the major histocompatibility complex (MHC) on chromosome 6, is crucial for the immune system in mediating adaptive and innate responses. HLA genes within this complex encode molecules that are essential for the regulation of immune activity and engaging in cellular defence mechanisms, interacting with components of both the innate and adaptive immune systems, including interactions with NK cells and crucial roles in presenting antigens to T cells (Klein and Sato, 2000; Medhasi and Chantratita, 2022).

HLA molecules, once expressed on the surface of cells, are pivotal in the recognition and presentation of antigens to T cells facilitating a targeted immune response (Medhasi and Chantratita, 2022). The variability within HLA genes is extensive, with thousands of alleles identified, highlighting their critical role in individual immune system diversity and disease susceptibility. This genetic diversity is catalogued in the IPD-IMGT/HLA database, underscoring the significance of HLA variations in immunogenetics, pharmacogenetics, and personalized medicine (Marsh and System, 2017; Robinson et al., 2020).

HLA class I molecules are ubiquitous on nucleated cells and present endogenously derived peptides to CD8^+^ T cells, which are essential for the elimination of infected or malignant cells. Conversely, HLA class II molecules, primarily expressed on professional antigen-presenting cells and certain activated T cells, present exogenously derived peptides to CD4^+^ T cells and thereby initiating and regulating adaptive immune responses (Traherne, 2008; Medhasi and Chantratita, 2022).

Unlike classical HLA genes, which primarily function in antigen presentation, non-classical HLA class I (HLA-E, HLA-F, HLA-G) and class II (HLA-DM, HLA-DO, HLA-DP) exhibit limited polymorphism and have more specialized roles in several regulations of immunity, like those that control NK cells (HLA-E), maternal–foetal (HLA-G) tolerance, regulation of peptide loading into classical class II (HLA-DM, HLA-DO), and fine-tuning immune responses rather than simple antigen presentation in maintaining immune homeostasis (Crux and Elahi, 2017).

The polymerase chain reaction (PCR)-based molecular techniques, the gold standard for HLA typing methods, are highly accurate but can be time-consuming and labour-intensive, particularly for comprehensive typing across multiple loci. Next-generation sequencing (NGS), however, offers significant improvements for such applications, enabling rapid, high-throughput sequencing of genomes, exomes, and targeted gene panels with the capacity for simultaneous processing of numerous samples. Technological choices in exome capture kits and sequencing parameters affect their application suitability, influencing genomic coverage breadth and depth. In this context, whole exome sequencing (WES) can be employ.ed in different configurations, tailored for clinical or research purposes. Clinical-grade WES adheres to stringent standards, providing reliable genetic data for diagnostic use in clinical settings, guided by regulatory compliances like CLIA and CAP. It employs high-coverage strategies to ensure detailed variant detection (Rehm et al., 2013; Aziz et al., 2015; Matthijs et al., 2016). In contrast, research-grade WES focuses on broader scientific inquiries with lower coverage, facilitating larger sample sizes and cost efficiency. It is suited for genetic association studies and diversity research, where depth below 100x is typical (Bamshad et al., 2011).

Ongoing advancements in NGS technology further increase HLA typing efficiency and resolution, despite challenges in handling the complex and highly polymorphic nature of HLA genes and the potential difficulty in resolving intricate sequence variations due to short read lengths (Dunn, 2011).

Several bioinformatics tools have been developed to call HLA alleles from NGS data, offering varying degrees of accuracy and resolution (Major et al., 2013; Kiyotani et al., 2017; Larjo et al., 2017; Matey-Hernandez et al., 2018; Chen et al., 2021; Liu et al., 2021; Thuesen et al., 2022; Xin et al., 2022; Claeys et al., 2023). These tools can be categorized into alignment-based and assembly-based methods, some of which are specifically designed for specific NGS data. Accurate polymorphism phasing is crucial for these tools, especially in the genetically complex HLA region. Most of the development, optimisation, and accuracy testing of these tools have been conducted using public data from the 1000 Genomes Project, which primarily focuses on HLA class I genes with a maximum of 2-field resolution and thereby may potentially introduce bias (Major et al., 2013; Larjo et al., 2017; Thuesen et al., 2022; Claeys et al., 2023).

While some tools demonstrate higher accuracy for HLA class I genes, they may not support HLA class II typing, or they may offer low, high, or allelic resolution levels. In clinical and research settings, it is often impractical to employ multiple tools; hence, a comprehensive tool that delivers high accuracy for the broadest range of HLA loci and resolution levels is necessary. Moreover, tools that are less computationally intensive and are able to yield quick results are favoured to facilitate timely and cost-effective analyses. This approach ensures efficient utilization of NGS data and resources, which are particularly important when considering the extensive applications of HLA typing. To identify the most effective tool for our study, we conducted a review of independent benchmarking studies, comparing the performance of various tools in typing both the HLA class I and class II genes. This analysis led to the selection of HLA-HD (Kawaguchi et al., 2017), based on its superior accuracy and resolution as well as on its computational efficiency and speed in delivering results across a wide range of HLA loci (details presented in Results section).

In the rapidly evolving field of HLA typing, the development and benchmarking of the available tools and algorithms have often relied on the same datasets from specific populations, a practice that may introduce bias. Additionally, such efforts typically have focused on a limited selection of HLA class I and II genes, potentially overlooking the accuracy and consistency of the tools in examining clinically and functionally relevant HLA genes. Notably, while alleles of HLA-DMA and -DMB are linked to rheumatoid arthritis (Morel et al., 2004), HLA-DOA, -DOB, and -DRA are associated with various diseases and autoimmune disorders (Okada et al., 2016; Li et al., 2020; Castelli et al., 2021; Nygård et al., 2021; Du et al., 2022; Liu and Hofman, 2022; Mei et al., 2022), there remains a knowledge gap concerning the HLA-J, -K, -L, and -V genes. These genes are essential to investigate for consistency and accuracy studies in HLA typing, as they have significant clinical relevance and represent areas where knowledge gaps exist, crucial for advancing future immunological research and understanding.

To address these limitations, our study undertakes the following two objectives: firstly, to validate the efficiency and comprehensiveness of the HLA-HD tool against the HLA-Twin tool and data generated by a commercial clinical-grade company; and secondly, to assess its consistency across diverse NGS datasets, including whole exomes, genomes, and HLA-targeted panels. By leveraging family trios and duplicate samples from underrepresented populations to investigate 29 HLA loci, we aim to provide a comprehensive approach that offers critical insights to the HLA research and clinical community. Achieving these objectives enables more informed choices in selecting HLA loci and determining resolution based on the characteristics of the NGS datasets from which HLA alleles are derived.

## Materials and methods

### Benchmarking studies on HLA typing tools

We evaluated published benchmarking studies to identify an HLA typing tool that offers comprehensive loci coverage and high accuracy for HLA class I and II genes using whole exome data of clinical-grade or research-grade quality applications. Our criteria focused on selecting a tool that maximizes HLA loci extraction from NGS datasets and on excluding tools with limited coverage.

This process ensured that the chosen tool meets the needs to achieve high accuracy, computational efficiency, and broad HLA loci coverage. For studies that scored HLA class I and II genes separately, we averaged their scores for a balanced evaluation, acknowledging that this might affect the overall ranking order. This analysis was crucial for determining the best-suited tool for our study needs.

### Study samples

**FIGURE 1:**
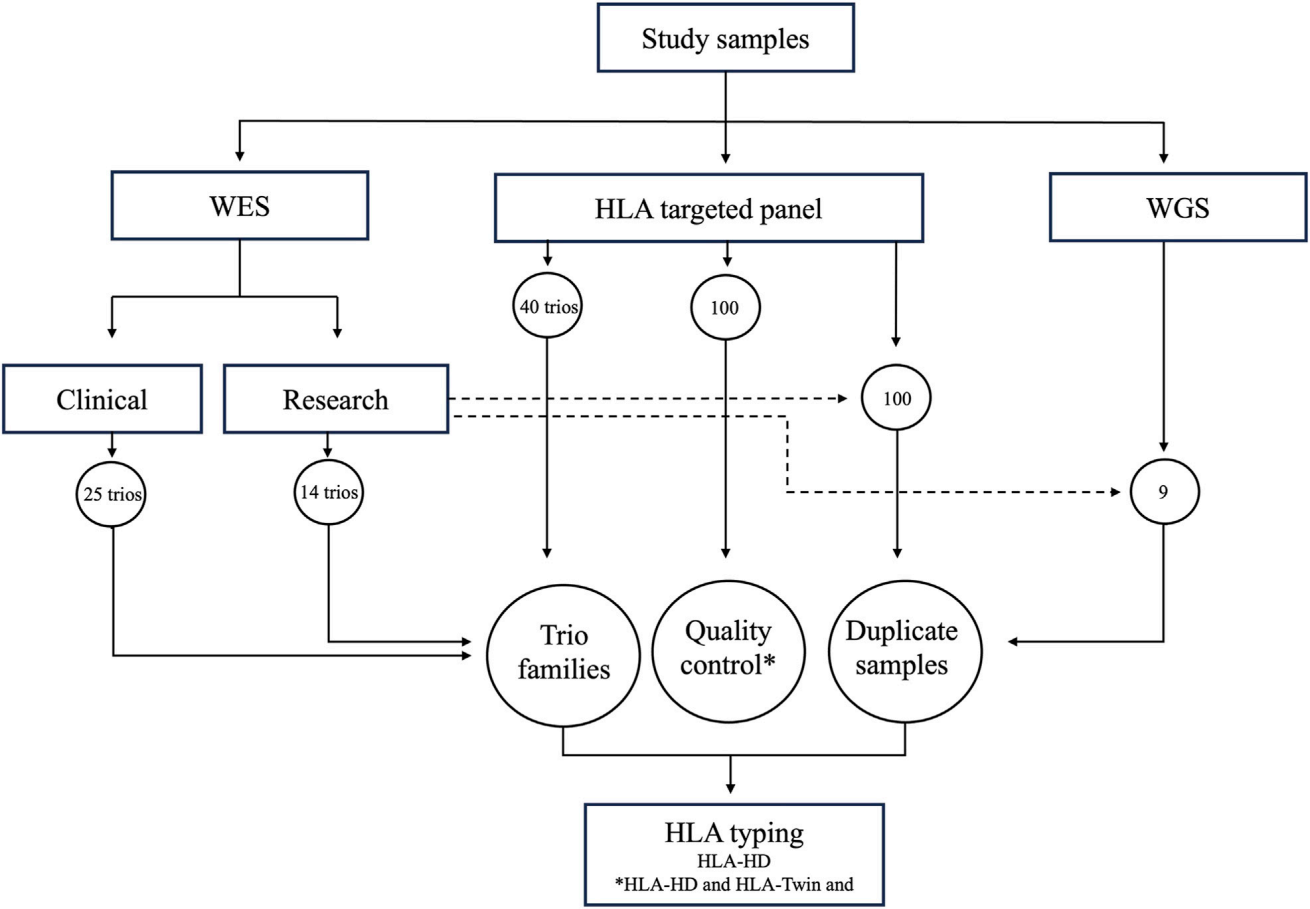
Study Workflow and Data Sampling Overview. Illustration of the varied NGS datasets used in the study. Whole exome sequencing data (WES) is categorized into (a) research data (14 family trios) and (2) clinical data (25 family trios). The study also includes targeted HLA panel data from 40 family trios and whole genome sequencing (WGS) data from nine individual genomes. Each dataset underwent HLA genotyping using the HLA-HD tool, with the HLA-Twin tool serving as a quality control measure and for evaluating HLA-HD’s accuracy.

The study protocol underwent review and received approval from the Ethical Review Committee of Dasman Diabetes Institute and was in accordance with the guidelines outlined in the Declaration of Helsinki. Family trios, consisting of parents and one child per family, were recruited at the Dasman Diabetes Institute in Kuwait. Prior to participating in the study, all adult participants provided signed informed consent forms. As regards minor participants, informed consent was obtained from their parents or legal guardians. Figure 1 provides a graphical overview of our study workflow, highlighting the range of NGS datasets examined in the study. Based on sequencing depth and read length, we classified the whole exome sequencing data into research (25 family trios) and clinical (14 family trios) groups. In addition, we analysed samples from 40 family trios through targeted HLA panel and nine samples from sporadic individuals through whole genome sequencing. Central to our analysis was incorporation of duplicate samples, with 100 overlapping the HLA targeted panel dataset and research-grade whole exome dataset, and nine overlapping the whole genome dataset and research-grade exome dataset. These duplicates, in conjunction with the family trios, were instrumental in affirming the consistency and reliability of the assessed HLA typing tool. Furthermore, we executed a thorough quality control measure, employing both HLA-HD and HLA-Twin tools on a subset of the HLA targeted panel dataset (100 samples), to verify the accuracy of the alleles determined by HLA-HD. We used HLA-Twin, optimized specifically for its proprietary HLA panel and known for diagnostic use, for initial quality control. Our primary HLA typing process relied on HLA-HD, which is known for its applicability to whole exome and whole genome datasets. Both the tools were applied concurrently, utilizing the same version of the IPD-IMGT/HLA database to minimize discrepancies in allele calling.

### Whole exome data

Genomic DNA was extracted from EDTA-anticoagulated peripheral blood samples using the QiAmp DNA blood mini kit following the manufacturer’s protocol. Illumina Novaseq 6,000 platform with Illumina DNA Prep with Enrichment kit was used to sequence 25 family trios (75 individuals). Additionally, the Illumina HiSeq 2,500 platform (Illumina, United States) was used to sequence the whole exomes of 14 family trios (42 individuals) using the Nextera capturing kit. To ensure consistent comparison, samples from 100 individuals that were sequenced on the Illumina platform were also sequenced using targeted HLA panel.

### Targeted HLA panel data

Total genomic DNA was extracted from blood samples using the QiAmp DNA blood mini kit, according to the manufacturer’s protocol. The Omixon Holotype HLA V3 kit (Omixon, Hungary) was used to generate DNA libraries and to sequence 11 loci (HLA-A, B, C, DRB1, DQA1, DQB1, DPA1, DPB1 and DRB3/4/5) from 40 family trios (120 individuals). A subset of the HLA targeted panel data (100 samples) was used for quality control and for analysis with duplicate samples that overlapped with research-grade whole exome dataset. The protocol involved long-range PCR amplification of the HLA genes using master mixes specific to each locus. The resulting PCR amplicon was quantitated and normalized using the QuantiFlour dsDNA system (Promega, United States). The amplicons were then subjected to enzymatic fragmentation, end repair, adenylation, and index ligation. The indexed libraries were purified using AMPure XP magnetic beads from Beckman (Coulter, United States) and their quantity was determined using a qubit fluorometer (Thermofisher Scientific, United States). Sequencing was performed on the Illumina Miseq platform (Illumina, United States), following the manufacturer’s instructions.

### Whole genome data

Sequence data of nine human whole genomes overlapping the research-grade exome dataset (8 Agilent SureSelect V5 and 1 Nextera capture kit) used in this study were obtained from previously published studies (Thareja et al., 2015; Fakhro et al., 2016). The NCBI SRA (Sequence Read Archive) accession numbers and identifiers of these samples are listed in Supplementary Table S1.

### HLA typing

The HLA-HD tool version 1.4.0 (Kawaguchi et al., 2017) was utilized to identify alleles in HLA genes using FastQ files from whole genome, exome, and targeted HLA panel sequencing data. The reads were compared to a reference panel from the IPD-IMGT/HLA database (3) version 3.46 build 2d19adf. The IPD-IMGT/HLA database can be accessed at https://www.ebi.ac.uk/ipd/imgt/hla/licence/. HLA-HD tools can type alleles at 29 HLA loci (HLA-A, B, C, DRB1, DQA1, DQB, DPA1, DPB1, DMA, DMB, DOA, DOB, DRA, DRB2, DRB3, DRB4, DRB5, DRB6, DRB7, DRB8, DRB9, E, F, G, H, J, K, L, and V) at a 3-field resolution (field 1:serological group, field 2:the specific HLA protein encoded, and field 3:synonymous nucleotide substitutions within the protein coding regions). In instances where there are insufficient reads to assign an allele for an HLA gene, the output is labelled as “Not typed.”

The HLA-HD and HLA-Twin version 3.1.1 tools were assessed for consistency in assigning resolutions by way of using the 100 duplicate samples sequenced on both the targeted HLA panel and the Illumina platform.

### HLA alleles consistency

We assessed success rates of HLA typing across various datasets, focusing on consistency at a 3-field resolution among family trios and duplicate samples, validated by concordant results from manual inspection by two investigators. We employed a Mendelian inheritance-based scoring system that awarded points for allele matches at each resolution field: one point for matches with both parents, half a point for a match with one parent, and zero for no match. “Not typed” alleles in duplicate samples were also scored to indicate potential absence or deletion. For “Not typed” alleles or dropout occurrences, we excluded from the count of families studied per locus from the trio consistency analysis to ensure accurate assessment. This adjustment was crucial, as incomplete data could hinder effective locus analysis. In contrast, duplicate samples were not excluded for “Not typed” results, facilitating direct comparisons of technology or tool effectiveness. Detailed information on families and duplicate samples with “Not typed” outcomes is provided in a Supplementary Table. Further, the DRB3/4/5 genes, due to their variable copy numbers and linkage to specific HLA-DRB1 alleles (Andersson et al., 1987; Andersson et al., 1994; Andersson, 1998), were treated as a single variation unit for consistency evaluation.

## Results

### Review of HLA typing tools validates HLA-HD’s superior performance

Results from evaluation of HLA typing tools for their accuracy in typing HLA genes are presented in Table 1. In our review, HLA-HD tool emerged as top performer, securing topmost rank in three benchmarking studies and second rank in two others. HLA*LA tool (Dilthey et al., 2019) was also notable for its high performance, ranking first in three studies and second in one study. HISAT-genotype tool (Kim et al., 2019) also proved to be reliable, achieving rank one in one study, rank two in two studies, and rank three in one study. Consistent performance across various evaluations highlights the precision and computational efficiency of HLA-HD, prompting us to make it our tool of choice. The utility of HLA*LA and HISAT-genotype tools was equally evident, with their effectiveness across diverse datasets and study conditions. These three tools are ranked further by comparing their computational efficiency with one another (Table 2). HLA-HD, which can type up to 29 HLA loci at a resolution of up to three fields, ranked as the most efficient in terms of memory usage and runtime per sample. HISAT-genotype, capable of typing 24 HLA loci with up to four fields of resolution, followed as the second most efficient tool. HLA*LA, though it types fewer loci (13 HLA genes) with up to three fields resolution, was found to be the third in line for efficiency.

**TABLE 1:** Ranking of HLA typing tools based on performance on whole exome datasets as reported in global studies.

Study	Rank 1	Rank 2	Rank 3
Lee et al. (2021)	HLA*LA	HLA-HD	PHLAT
Bauer et al. (2018)	PHLAT	HLA-VBSeq	HLAminer
Yu et al. (2021)	seq2HLA	HISAT-genotype	HLA forest
Liu et al. (2021)	HLA-HD, HISAT-genotype	HLAscan	HLAminer
Chen et al. (2021)	HLA-HD	HLA*LA	HISAT-genotype
Claeys et al. (2023)	HLA*LA	HLA-HD	xHLA
Thuesen et al. (2022)	HLA*LA	HISAT-genotype	Kourami
Xin et al. (2022)	HLA-HD	xHLA	HLAscan

**TABLE 2:** Comparison of computational efficiency and capabilities of top HLA typing tools on whole exome data across studies.

Tool	Number of HLA loci typed	Resolution level	Ranking in terms of memory usage	Ranking in terms of runtime efficiency
HLA-HD	29	Up to 3 fields	Best (1st)	Best (1st)
HISAT-genotype	24	Up to 4 fields	Good (2nd)	Good (2nd)
HLA*LA	13	Up to 3 fields	Fair (3rd)	Fair (3rd)
Studies	Thuesen et al., 2022; Claeys et al., 2023			

### Characteristics of the NGS dataset and their impact on HLA typing performance

To ensure the integrity and reliability of our HLA typing analysis, we distinguished clinical-grade exomes from research-grade exomes based on sequencing depth and read length. Research-grade exomes are those sequenced with paired-end reads of 100 bp and a coverage ranging from 42–76X, and clinical-grade exomes are those with 150 bp reads and 96–260X coverage. This classification helped in underscoring the significance of high-quality sequencing data for accurate HLA allele calling.

Our evaluation extended to assessing the impact of the different exome capture kits on HLA typing. Specifically, we observed that datasets sequenced using Agilent SureSelect V4 kits from projects SRP060765, SRP061943, and SRP061463, and the whole genome dataset from the Illumina HiSeq 2000 platform, utilizing the TruSeq library kit (John et al., 2015), failed to consistently produce typing results for nearly all the 29 HLA loci, indicating ‘Not Typed’ outcomes across both HLA class I and II genes. This pattern of pervasive typing failures suggests a compatibility issue with earlier sequencing technologies, likely stemming from insufficient read depth, and specificity of capture kits for HLA regions.

Considering these challenges and the need to maintain the analytical robustness of our study, we made the informed decision to exclude datasets sequenced with Agilent SureSelect V4 and Illumina HiSeq 2000. This approach allowed us to use only the data obtained from newer and more compatible sequencing technologies, enhancing the overall quality and applicability of our findings.

### Success rate of HLA typing with HLA-HD across NGS datasets

**FIGURE 2:**
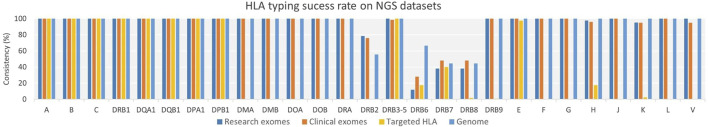
HLA Typing Success Rate for NGS Datasets Across Varying Genes and Resolutions. NGS datasets achieved a very high success rate of HLA typing for classical HLA class I and class II genes. However, the datasets encountered difficulties in calling alleles for the DRB2 and DRB6–8 genes. Additionally, the genomes and exomes (both research and clinical) datasets achieved a high success rate of HLA typing for non-classical HLA class I genes (E, F, G, J, K, L, and V) and class II genes (DMA, DMB, DOA, and DOB).

We conducted tests to evaluate the performance of HLA-HD in HLA typing across various NGS datasets, including clinical-grade and research-grade exomes, targeted HLA panel (Omixon), and genomes. Our findings demonstrated that HLA-HD achieved high success rates in calling HLA alleles, primarily at the 3-field resolution, across different NGS datasets. Notably, it displayed a 100% success rate for the classical HLA class I genes (HLA-A, -B and -C) and most of the class II genes (HLA-DR, -DQ, and -DP; Figure 2). As regards the DRB genes, the success rate of HLA typing was high when the DRB3/4/5 genes were treated as a single region, however, the other DRB genes such as DRB2, and DRB6-8 had low success rates of allele typing. This can be attributed to their paralogous relationship with the DRB1 gene and shared DNA sequences (Zhang et al., 2017) which may complicate the probabilistic model for estimation of reads assigned by HLA-HD between the input data and the IMGT/HLA database, resulting in a “Not typed” output.

Furthermore, the discrepancy in success call rates between the exomes of research- and clinical-grade manifested in these specific genes. As expected, the targeted HLA panel dataset exhibited either failure or very low success rates in calling alleles for untargeted HLA genes (HLA-DMA, -DMB, -DOA, -DOB, -DRA, -F, -G, -H, -J, -K, -L, and -V), when compared to the genome and exome datasets. However, it displayed success in typing alleles for HLA-E loci, despite not being explicitly advocated for such purposes.

### Comparative consistency analysis of HLA typing by HLA-HD and HLA-Twin tools on HLA panel dataset

**FIGURE 3:**
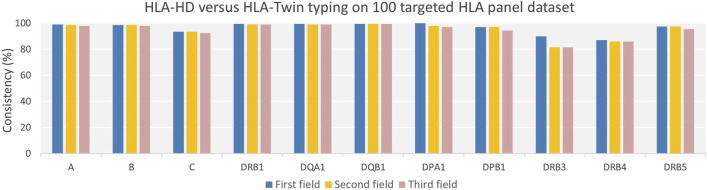
Consistency of HLA typing of Targeted HLA Panel Dataset by HLA-HD versus HLA-Twin Tools Across Varying Genes and Resolutions. The chart presents the degree of agreement between HLA-HD and HLA-Twin tools for HLA typing across diverse genes and resolutions, represented by consistency percentages. While high consistency percentages are evident for all HLA genes across various resolutions, it is less pronounced for the DRB3/4/5 genes.

Our comparative analysis of HLA typing consistency, utilizing the HLA-HD and HLA-Twin tools across a dataset of 100 targeted HLA panel samples, demonstrated high concordance across 11 HLA loci, with average concordance percentages of 96.45%, 95.27%, and 94.59% at the first, second, and third field resolutions, respectively (Figure 3). However, significant discrepancies were observed in the DRB gene regions, notably in the DRB3/4/5 region. We further analysed the discrepancies by reviewing raw typing data for all HLA loci, as detailed in Supplementary Table S2, which highlighted occurrence of untyped alleles in HLA-Twin’s automated analysis. For DRB3, HLA-Twin’s automated process failed to identify alleles in eight individuals, seven of whom were found to have homozygous alleles, notably three with the HLA-DRB3*02:02:20 variant. In the case of DRB4, HLA-Twin’s automated typing did not recognize alleles in 13 individuals, where 12 presented with homozygous alleles, including six carrying the HLA-DRB4*01:03 variant and 5 with HLA-DRB4*01:138. Further analysis revealed a higher prevalence of HLA-C*07 and HLA-C*04 alleles in samples where HLA-Twin provided no automated results.

### Consistency analysis in family trios

Using Mendel’s laws of inheritance, we assessed the consistency of HLA alleles inherited from parents by children in two trio datasets: exomes (of research- and clinical-grade) and targeted HLA panel. We calculated the consistency percentage of HLA alleles across various HLA genes and resolutions, considering alleles that were successfully typed in both parents and children.

In our analysis of HLA typing by HLA-HD tool of the dataset comprising research-grade exomes of 14 family trios, we focused on percentage of trios with consistent HLA typing between parents and child (Table 3 and Supplementary Table S3). HLA-HD achieved average consistency percentages of 97.18%, 91.27%, and 90.21% across 29 HLA loci at the first, second, and third field resolutions, respectively, with an overall average consistency of 92.89% across all resolutions. The DPB1 locus exhibited the lowest consistency percentage among HLA genes across evaluated field resolutions, reaching only 57.14%. This was followed by lower consistency percentages at the second and third field resolutions in the HLA-A locus and at the third field resolution in HLA-G. Additionally, limited-to-no successful allele typing between parents and children was observed at the DRB6 locus in the research-grade exome family trios.

**TABLE 3:** Consistency of HLA typing for research grade exome trios across varying genes and resolutions.

Genes	First field (%)	Second field (%)	Third field (%)	Families studied
A	96.43	67.86	64.29	14
B	92.86	92.86	92.86	14
C	96.43	89.29	89.29	14
DRB1	89.29	82.14	82.14	14
DQA1	100	96.43	96.43	14
DQB1	96.43	78.57	78.57	14
DPA1	100	82.14	78.57	14
DPB1	57.14	57.14	57.14	14
DMA	100	100	100	14
DMB	100	82.14	82.14	14
DOA	100	100	92.86	14
DOB	100	100	100	14
DRA	100	100	96.43	14
DRB2	100	100	100	9
DRB3	100	91.67	87.5	12
DRB4	100	90	90	5
DRB5	100	100	100	2
DRB6	NA	NA	NA	0
DRB7	100	100	100	4
DRB9	100	100	100	4
DRB9	100	100	100	14
E	96.43	85.71	85.71	14
F	100	96.43	96.43	14
G	100	78.57	75	14
H	96.15	92.31	92.31	13
J	100	100	100	14
K	100	92.31	92.31	13
L	100	100	96.15	14
V	100	100	100	14

Further investigation into the DPB1 locus inconsistencies, as detailed in Supplementary Table S3, revealed that three probands had alleles inconsistent with both of their parents, while six probands showed alleles consistent with only one parent. Across the analysed nine trios, no more than two trios had family members with homozygous alleles, and no more than three trios had family members with a specific allele. These factors did not appear to be the primary causes of the observed inconsistency. Notably, a common pattern identified in seven out of these nine trios was that at least one member had an allele typed at only two fields resolution, suggesting insufficient coverage as a potential contributing factor to the discrepancies observed. In investigating the HLA-A locus at the second field resolution, a similar pattern emerged where six trios had at least one family member with alleles typed at only two fields resolution, further suggesting that insufficient coverage might be a significant issue affecting the consistency of HLA typing.

Further analysis of the HLA-G locus at the third field resolution revealed two trios with a family member having both alleles at the third field resolution differing from both parents, and three probands with one inconsistent allele at the third field with one of the parents. No specific alleles, either homozygous or not, was repeated in more than two trios, indicating that polymorphism phasing at the HLA-G locus could be the issue contributing to these inconsistencies.

When evaluating the consistency percentage of alleles across 29 HLA loci in the dataset of 25 clinical-grade exomes from family trios, the HLA-HD tool achieved average consistency percentages of 99.11% at the first field resolution, 98% at the second, and 97.42% at the third (Table 4 and Supplementary Table S4). Inconsistencies in HLA alleles within the clinical-grade exome dataset were minimal across evaluated field resolutions and were mainly observed at the HLA-H locus. Notably, there were limited-to-no successfully typed alleles between parents and children at the DRB6 loci in the clinical-grade exome family trios. Further investigation into the HLA-H locus inconsistencies revealed specific patterns. Firstly, only two trios had family members with untyped alleles, indicating isolated incidents of typing failures. Secondly, in six trios, probands matched only one of their parent’s alleles, which were observed to be homozygous in the probands. This pattern might indicate a homozygous typing issue by HLA-HD at the HLA-H locus, potentially contributing to the observed inconsistencies.

**TABLE 4:** Consistency of HLA typing for clinical grade exome family trios across varying genes and resolutions.

Genes	First field (%)	Second field (%)>	Third field (%)	Families studied
A	100	100	98	25
B	96.15	94.87	94.87	25
C	98	98	98	25
DRB1	98	98	98	25
DQA1	100	100	100	25
DQB1	100	100	98	25
DPA1	98	98	98	25
DPB1	98	98	98	25
DMA	100	100	100	25
DMB	100	88	88	25
DOA	100	100	100	25
DOB	100	98	96	25
DRA	100	100	100	25
DRB2	100	100	100	11
DRB3	100	100	100	18
DRB4	100	100	100	13
DRB5	100	100	100	3
DRB6	NA	NA	NA	0
DRB7	100	100	100	5
DRB8	100	100	100	5
DRB9	100	96	96	25
E	100	100	96	25
F	100	100	100	25
G	100	100	96	25
H	86.96	86.96	84.78	23
J	100	100	100	25
K	100	88.1	88.1	21
L	100	100	100	25
V	100	100	100	23

In the dataset of 40 family trios sequenced using a targeted HLA panel, the HLA-HD tool demonstrated average consistency percentages of 98.97%, 97.60%, and 97.47% at the first, second, and third field resolutions, respectively, across 11 HLA loci. The average consistency percentage for all the HLA genes was 98% across evaluated field resolutions (Table 5 and Supplementary Table S5), surpassing the performance of research-grade exome trio datasets and matching that of the clinical-grade exome trio datasets. Inconsistencies in HLA alleles in the targeted HLA panel dataset were very minimal across evaluated field resolutions and were mainly observed at the DRB3-4 loci, which showed the lowest success rates in allele typing between the parents and children. These findings suggest that the custom design of sequence capture can positively impact the consistency of HLA allele typing.

**TABLE 5:** Consistency of HLA typing for targeted HLA panel trios across varying genes and resolutions.

Genes	First field (%)	Second field (%)	Third field (%)	Families studied
A	100	98.75	98.75	40
B	100	100	100	40
C	100	100	100	40
DRB1	100	100	100	40
DQA1	100	100	100	40
DQB1	100	100	100	40
DPA1	100	100	100	40
DPB1	97.5	97.5	97.5	40
DRB3	94.45	90.28	88.89	24
DRB4	96.78	87.10	87.10	22
DRB5	100	100	100	20

### Duplicate sample analysis for HLA alleles consistency

**FIGURE 4:**
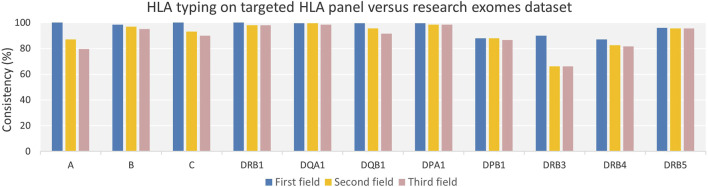
Consistency of HLA Typing for Duplicate Samples from Targeted HLA Panel and Research Exomes Across Varying Genes and Resolutions. The consistency percentage rate across classical HLA class I and class II genes were high across evaluated field resolutions among duplicate samples from targeted HLA panel and research exome dataset. HLA allele consistency decreased gradually with increasing HLA resolution from the first to second, and second to third field levels.

We used 100 duplicate samples from two NGS datasets, namely, targeted HLA panels and research-grade exomes, as an alternative approach to assess the consistency of HLA alleles by the HLA-HD tool. The consistency percentage of HLA alleles across various genes and resolutions was calculated (Figure 4 and Supplementary Table S6). The average consistency rates across 11 HLA loci were 92% for all evaluated fields of resolution, detailed as 96.18% at the first, 90.95% at the second, and 89.14% at the third field resolution, respectively.

Our analysis revealed a gradual decline in HLA allele consistency as the resolution progressed from the first field to the second, and from the second to the third field resolutions. Furthermore, higher inconsistency was noted in HLA-DPB1 and DRB3/4 genes across evaluated field resolutions, which exhibited the lowest success rates in allele typing between duplicate samples.

Further investigation into the inconsistencies at the DPB1 locus (Supplementary Table S6) revealed that most inconsistencies stemmed from typing one or both alleles at only two fields resolution when using research-grade exome data, compared to three fields resolution using targeted HLA panel. This suggests a coverage issue, which may also explain why seven samples did not match exactly between the two datasets; these were typed at two fields in research-grade exomes without a specific allele or allele combination being identifiable.

Regarding the DRB3/4 locus, inconsistencies were observed in samples that had ‘Not typed’ alleles in either one of the datasets. Furthermore, the inconsistency in the majority of DRB3 alleles, particularly at the second and third field resolutions, was due to the observation that alleles from research-grade exome samples were only typed up to the second field, whereas data from the targeted HLA panel provided three field resolutions. A recurrent allele in more than 40 samples was typed as HLA-DRB3*02:96 b y research-grade exome data, while it was identified as HLA-DRB3*02:02:01 in the targeted HLA panel.

**FIGURE 5:**
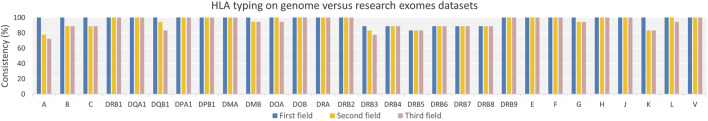
Consistency of HLA Typing for Duplicate Samples from Genomes and Research Exomes Across Varying Genes and Resolutions. The consistency percentage rate across HLA genes was very high across evaluated field resolutions among duplicate samples from genomes and exomes research dataset. The HLA alleles consistency decreased gradually as HLA resolution increased from the first to the second field levels.

The average consistency percentages across 29 HLA loci for duplicate samples from nine whole genome and research-grade exome datasets were 97.51%, 94.64%, and 93.49% at the first, second, and third field resolutions, respectively, with an overall average consistency across all fields of resolution of 95.21% (Figure 5 and Supplementary Table S7). The precision of HLA typing consistency gradually decreased as the resolution increased from the first field to the second, particularly for HLA-A, B, and C. This decline could be attributed to the whole genome data often yielding calls at only two fields, in contrast to the three fields resolution provided by research-grade exome data. Moreover, whole genome samples showed a few instances of unsuccessful HLA allele typing at the DRB genes. For example, five samples from whole genome data had “Not typed” alleles at DRB genes, compared to only one sample from research-grade exome data, as detailed in Supplementary Table S7.

## Discussion

This study evaluates the consistency of HLA typing, covering an extensive range of HLA genes and resolutions, using a comprehensive and efficient tool, across a spectrum of NGS datasets that include whole genome, research-as well as clinical-grade whole exome, and targeted HLA panel. HLA typing was performed using HLA-HD, which can type up to 29 HLA loci at a resolution of up to three fields.

The proficiency of the HLA-HD tool in identifying HLA alleles, especially in both HLA class I and II genes, is consistently demonstrated across these datasets. It is worth noting that many of these datasets were not initially tailored for HLA typing. This underscores an essential insight: existing whole genome and exome datasets, even if initially curated for different clinical or research purposes, can be adaptively repurposed. Such datasets then become invaluable secondary resources for endeavours in population genetics (Gourraud et al., 2014), pharmacogenetics (Dashti et al., 2022), and disease association studies (Haris et al., 2021; Afyouni et al., 2022).

Our study demonstrates how the choice of NGS capturing kit, along with coverage depth and read length, significantly influences the accuracy and consistency of HLA allele typing across various genes and resolutions. The observed improvements with optimized capturing kits, increased coverage, and longer reads align with findings from previous studies, underscoring the importance of these parameters in achieving precise HLA genotype accuracy (Major et al., 2013; Liu et al., 2021; Thuesen et al., 2022).

Quality control comparison of HLA-HD and HLA-Twin tools revealed high concordance, achieving an overall average consistency rate of 95.44% across all resolutions for 11 HLA loci. However, we also identified notable discrepancies, particularly with alleles such as HLA-C*07 and HLA-C*04, which were consistently identified by HLA-HD but not by HLA-Twin. This pattern suggests a potential specificity issue in HLA-Twin’s ability to type certain alleles, notably exemplified by the homozygous alleles DRB3*02:02:20 and DRB4*01:03. This suggests that the discrepancies might be specific to certain alleles and/or their homozygous state. Nevertheless, HLA-Twin typically flags untyped alleles, allowing for manual intervention—an important feature in diagnostic laboratories to ensure accurate allele typing, a feature that HLA-HD does not offer. However, this step of manual intervention was not undertaken in our comparison to maintain objectivity and avoid subjective parameter adjustments.

Furthermore, the well-documented complexity of the DRB gene region, aligning with our observations, presents specific challenges in NGS HLA typing, where technical and biological factors significantly complicate allele calling in these problematic loci. Specialized PCR techniques and *in silico* approaches have been proposed to mitigate the ambiguity of HLA allele calling for the DRB2-5 loci (Ozakiet al., 2014; Zhang et al., 2017). Achieving unambiguous allele calls in these regions is crucial, given their implication in the risk of several autoimmune disorders (Zamani et al., 2001; Odani, et al., 2012; Erlich et al., 2013; Zhao et al., 2016) and histocompatibility (Detrait et al., 2015; 3 Fernandez-Vina et al., 2013). Thus, accuracy and consistency studies for these genes are beneficial, enabling informed decisions about the selection of HLA typing tools.

Building on the established need for precise HLA typing, particularly for less explored HLA genes, our study includes a consistency analysis in family trios of the research-grade exome dataset across 29 HLA loci. Based on our consistency metric score, we achieved an average consistency rate of 92.89% across all resolutions for these loci. High consistency rates were observed at the DRB3/4/5 loci for trios with typed alleles across all members. Nonetheless, a few family trios were excluded from our study due to the absence of typed alleles (‘Not typed’) in at least one family member. Given the specific inheritance patterns for DRB3/4/5 linked to certain HLA-DRB1 alleles, as delineated by Andersson et al. (Andersson et al., 1987; Andersson et al., 1994; Andersson, 1998), our analysis focused only on instances where complete allele typing was available for all family members. This focus includes detailed examination of inheritance scenarios peculiar to these loci: cases where alleles are inherited solely from one parent, and instances reflecting the absence of one DRB3/4/5 allele locus in the event of homozygosity at another DRB3/4/5 allele locus.

Moreover, our investigation highlighted specific loci, notably HLA-A, DPB1, and HLA-G, which exhibited lower consistency percentages at higher field resolutions. This pattern indicates a reduction in allele calling accuracy with increased resolution, likely due to the inherent limitations of NGS technology, such as capturing kit capabilities, coverage depth, and read length, rather than specific allele combinations or homozygosity. This conclusion is further supported by our comparative analysis of duplicate samples from the research-grade exome and HLA targeted panel datasets, as well as the genome dataset. Despite these identified challenges, the overall consistency within the research-grade exome dataset was satisfactory across all HLA genes and resolutions. Importantly, the first field resolution of HLA alleles remains a valuable tool for identifying genetic susceptibility or protection against certain diseases (Altfeld et al., 2003; Bowness, 2015).

Extending the analysis to family trios within the clinical-grade exome dataset resulted in very high consistency of HLA allele typing across 29 HLA loci. The overall average consistency of 98% for all field resolutions, surpassed that of the research-grade exome dataset and achieved parity with the HLA target panel dataset. Moreover, the average consistency of HLA alleles at the HLA-DRB3/4/5 loci in the clinical-grade exome trio dataset slightly exceeded that of the HLA targeted panel trio dataset. Notably, at the HLA-DRB3/4/5 loci, consistency in the clinical-grade exome dataset slightly exceeded that observed in the HLA targeted panel dataset, emphasizing the value of clinical-grade exomes for clinical applications involving HLA alleles (Liu et al., 2021; Thuesen et al., 2022). For example, discrepancies in the antigen recognition domain of HLA-DRB3/4/5 genes have been linked to poorer overall survival and increased non-relapse mortality in transplant pairs that would otherwise be considered a 10/10 HLA match (Ducreux et al., 2018; Tsamadou et al., 2021). Additionally, the clinical-grade exome dataset can be beneficial for research purposes, especially in successfully typing HLA alleles at less explored HLA genes that have been successfully identified.

The observed improvement in consistency across most of the 29 HLA loci using the dataset of clinical-grade exomes, underscores the importance of enhanced NGS capturing kit (Nextera), read depth, and read length as key factors in the reliability of allele typing. Nevertheless, a detailed analysis of the clinical-grade exome dataset uncovered instances of untyped alleles at the HLA-H locus and scenarios where probands matched only one parent’s alleles, often homozygous. These issues may arise from HLA-HD’s challenges in distinguishing heterozygous from homozygous alleles at this locus, potentially compounded by the Nextera kit’s capture design inadequately covering the HLA-H locus. In contrast, research-grade exome trios, sequenced with the older Illumina TruSeq kit exhibiting lower coverage, demonstrated higher consistency, suggesting capture kit choice to significantly affect typing consistency. Additionally, the larger sample size in clinical-grade exome dataset compared to research-grade exome dataset points out that sample size may also impact the detection of typing inconsistencies at the HLA-H locus.

The analysis of the targeted HLA panel trio dataset reveals high consistency across 11 HLA loci, highlighting its suitability for clinical applications due to its cost-effectiveness compared to the other NGS datasets. This panel is specifically tailored for the classical class I and II genes (11 HLA loci). Such findings are further substantiated by comparisons between duplicate samples from the targeted HLA panel and the research exome dataset, where we noted significantly improved typing accuracy and reliability at complex HLA loci and at higher resolution levels, especially for the DRB3/4/5 and DPB1 loci. Nonetheless, minor inconsistencies and typing challenges at the DRB3/4/5 loci may stem from the inherent complexity of these regions. However, when budgetary considerations are secondary, genome and exome datasets (of both clinical- and research-grade) provide the ability to type HLA alleles beyond the classical Class I and II genes. Despite these genes exhibiting fewer allelic polymorphisms and having less understood biological functions, they show remarkable consistency in our NGS datasets, making them valuable for both clinical and research applications.

An interesting finding from the comparison of duplicate samples between whole genome and research-grade exome datasets is the presence of untyped alleles, and alleles limited to second field resolution in genome data compared to exome data. This suggests that the full sequence of HLA regions by whole genome sequencing may not be as crucial as the read coverage achieved through research-grade exome sequencing for the consistency of HLA allele calling. It also implies that the HLA-HD tool may be better optimized for exome data than genome data. Furthermore, we observed that early genome sequencing data obtained using earlier Illumina platforms are not suitable for HLA typing, a conclusion that is consistent with findings from another study (Major et al., 2013). Similarly, earlier exome capture kits, such as Agilent SureSelect V4, are also found to be less effective for HLA typing.

The inconsistencies observed in the typing of DRB3, DRB4, and DRB5 alleles across evaluated NGS datasets, including trios and duplicates analysis, highlight significant challenges stemming from the paralogous nature and sequence similarity of these genes with DRB1 (Zhang et al., 2017; Ozakiet al., 2014). Their generally lower expression levels further diminish the detectability of relevant reads, compounding the inherent difficulties faced by short-read NGS technologies in precise read mapping and assignment within these regions. Notably, the majority of benchmarking studies and HLA typing tools (Major et al., 2013; Kiyotani et al., 2017; Larjo et al., 2017; Matey-Hernandez et al., 2018; Liu et al., 2021; Thuesen et al., 2022; Xin et al., 2022; Claeys et al., 2023) have excluded these genes, likely due to these complexities, underscoring a critical gap in current typing methodologies. To address these challenges, it is imperative to refine HLA typing approaches specifically tailored for these genes, potentially involving the development of dedicated algorithms, the adoption of longer-read sequencing technologies or specialized bioinformatics tools adept at managing complex genomic regions, and the expansion of the IMGT/HLA database to enhance imputation capabilities to accurately fine-map the MHC region. Additionally, revising the current capture design of exome sequencing, whole-genome sequencing, and targeted HLA region sequencing to better accommodate the complexities of these loci could lead to more reliable detection and typing of these alleles, ultimately improving our understanding of their role in immune function and advancing the field of HLA typing.

The current study has certain limitations due to its retrospective design. One limitation is the absence of a gold standard method to validate the concordance of typed alleles across different HLA genes and resolutions. However, assessing consistency provides a realistic measure of reproducibility and reflects the reliability of different NGS datasets on HLA typing. Another constraint is the lack of access to a genome family trio dataset or duplicate sample comparisons between clinical-grade exomes and HLA target panel or genomes, which could have further enriched our study. We excluded higher field resolution from our analysis due to the extensive time required to analyse it in large sample sets (Thuesen et al., 2022; Claeys et al., 2023). While we did evaluate the consistency between the HLA-HD tool and the HLA-Twin tool on a targeted HLA panel dataset, our primary reliance was on a single NGS-based typing tool known for its high performance (Chen et al., 2021; Xin et al., 2022); however, incorporating other *in silico* tools would offer a more comprehensive comparison.

Moreover, we used the HLA-HD tool, which is confined to alleles in the IPD-IMGT/HLA database and cannot detect novel alleles, limiting the diversity of alleles analysed. In contrast, tools such as HISAT-genotype (Kim et al., 2019) can identify novel alleles by adapting to variations absent in standard references. Since our study focused on evaluating the consistency of HLA typing, this limitation should not impact our findings.

Additionally, our findings reveal that while HLA allele segregation in family trios generally shows high consistency, it does not achieve complete concordance. It is important to note this observation, as the precision required for HLA polymorphism admits little room for error, particularly in classical class I and class II HLA genes. Despite advancements, current NGS tools do not achieve complete accuracy in HLA allele concordance using exome data and trio families (Bauer et al., 2018; Chen et al., 2021; Lee et al., 2021; Liu et al., 2021; Yu et al., 2021; Thuesen et al., 2022; Xin et al., 2022; Claeys et al., 2023). These findings highlight the need for further enhancements in algorithms and the expansion of reference databases such as IPD-IMGT/HLA to bolster imputation capabilities.

Finally, the sample size, inherent to the trio family study design and our reliance on retrospective datasets, represents a limitation. We addressed this by using percentage-based metrics to maintain comparability and robustness of our findings across limited sample sizes.

## Conclusion

Our study validates HLA-HD as the most comprehensive bioinformatics tool for HLA typing, covering the broadest range of loci compared to other tools across a spectrum of NGS datasets. Demonstrating its reliability and high consistency in allele identification, this approach streamlines the HLA typing process to align with the practical needs of both research and clinical settings, emphasizing cost-effectiveness and computational efficiency. By showcasing HLA-HD’s compatibility with diverse NGS datasets, our findings advocate for its broad application in immunogenetics research and personalized medicine, suggesting its potential as a standardized tool for accurate HLA allele detection.

The insights gained from our analysis underline the importance of NGS read depth and length, as well as the selection of exome capture kits, in influencing the consistency rates of HLA typing. These considerations are crucial for researchers assessing the feasibility of utilizing available NGS datasets in studies relating to calling HLA alleles. For future studies, the integration of advanced sequencing technologies, including long-read sequencing, holds promise for enhancing the accuracy and consistency of HLA typing further.

## Data Availability

The datasets presented in this study can be found in online repositories. The names of the repository/repositories and accession number(s) can be found in the article/Supplementary Material.
